# Delta radiomic features improve prediction for lung cancer incidence: A nested case–control analysis of the National Lung Screening Trial

**DOI:** 10.1002/cam4.1852

**Published:** 2018-12-01

**Authors:** Dmitry Cherezov, Samuel H. Hawkins, Dmitry B. Goldgof, Lawrence O. Hall, Ying Liu, Qian Li, Yoganand Balagurunathan, Robert J. Gillies, Matthew B. Schabath

**Affiliations:** ^1^ Department of Computer Sciences and Engineering University of South Florida Tampa Florida; ^2^ Department of Cancer Physiology H. Lee Moffitt Cancer Center and Research Institute Tampa Florida; ^3^ Department of Radiology Tianjin Medical University Cancer Institute and Hospital, National Clinical Research Center of Cancer Key Laboratory of Cancer Prevention and Therapy Tianjin China; ^4^ Department of Cancer Epidemiology H. Lee Moffitt Cancer Center and Research Institute Tampa Florida

**Keywords:** early detection, lung cancer screening, National Lung Screening Trial, quantitative imaging, Radiomics

## Abstract

**Background:**

Current guidelines for lung cancer screening increased a positive scan threshold to a 6 mm longest diameter. We extracted radiomic features from baseline and follow‐up screens and performed size‐specific analyses to predict lung cancer incidence using three nodule size classes (<6 mm [small], 6‐16 mm [intermediate], and ≥16 mm [large]).

**Methods:**

We extracted 219 features from baseline (T0) nodules and 219 delta features which are the change from T0 to first follow‐up (T1). Nodules were identified for 160 incidence cases diagnosed with lung cancer at T1 or second follow‐up screen (T2) and for 307 nodule‐positive controls that had three consecutive positive screens not diagnosed as lung cancer. The cases and controls were split into training and test cohorts; classifier models were used to identify the most predictive features.

**Results:**

The final models revealed modest improvements for baseline and delta features when compared to only baseline features. The AUROCs for small‐ and intermediate‐sized nodules were 0.83 (95% CI 0.76‐0.90) and 0.76 (95% CI 0.71‐0.81) for baseline‐only radiomic features, respectively, and 0.84 (95% CI 0.77‐0.90) and 0.84 (95% CI 0.80‐0.88) for baseline and delta features, respectively. When intermediate and large nodules were combined, the AUROC for baseline‐only features was 0.80 (95% CI 0.76‐0.84) compared with 0.86 (95% CI 0.83‐0.89) for baseline and delta features.

**Conclusions:**

We found modest improvements in predicting lung cancer incidence by combining baseline and delta radiomics. Radiomics could be used to improve current size‐based screening guidelines.

## 1 INTRODUCTION

The National Lung Screening Trial (NLST) compared low‐dose helical computed tomography (LDCT) vs standard chest radiography for three annual screens and revealed a 20% relative reduction in lung cancer mortality among participants screened with LDCT.^1^, ^2^, ^3^ In the LDCT arm, screen‐detected incident lung cancers were found 2.7‐fold higher associated with a stage shift from late stage to more early‐stage lung cancers and exhibited improved 5‐year survival compared with prevalence cancers diagnosed at baseline.^3^, ^4^ Despite the benefits associated with lung cancer screening, LDCT imaging is associated with a high rate of detection of indeterminate pulmonary nodules (IPNs) of which only a fraction are diagnosed as lung cancer. In the NLST, 96.4% of the positive LDCT screens were false positives/IPNs. Though clinical guidelines^5^, ^6^, ^7^ provide for the evaluation and follow‐up of nodules, there are no validated clinical decision tools to predict lung cancer risk and probability of cancer development. Ideally, an efficient and accurate noninvasive approach should be developed as a clinical decision tool for radiologists and pulmonologists to better manage nodules, especially IPNs, in the lung cancer screening setting.

Radiomics is the process of converting standard‐of‐care digital medical images into quantitative image‐based feature data that can be subsequently analyzed using conventional biostatistics and machine learning methods.^6^ With high‐throughput computing, it is now possible to rapidly extract radiomic features from a region of interest that quantify size, shape, intensity, and texture of the region of interest. As radiomic features are likely capturing biological and pathophysiology information of the region of interest,^6^ radiomics have the potential to provide a rapid and accurate noninvasive approach to better manage pulmonary nodules detected by LDCT in the lung cancer screening setting.

In this study we conducted a nested case–control analysis of the NLST, using training and test sets, to identify radiomic features that are predictive of lung cancer incidence. We analyzed robust and reproducible radiomic features^8^ from baseline (T0)‐positive screens in the LDCT arm of the NLST to identify radiomic models that predict lung cancer incidence in the first (T1) and second (T2) follow‐up screening intervals. Moreover, we also included delta radiomic features to determine whether changes in the nodules over time from T0 to T1 improve predicting lung cancer incidence. Current guideline algorithms for managing LDCT‐detected solid and subsolid nodules are largely based on size, specifically longest diameter. As recommended by the National Comprehensive Cancer Network (NCCN)^5^ and the American College of Radiology (ACR),^6^, ^7^ the current cutoff size for assessing lung nodules increased to 6 mm rather than the 4 mm originally used in the NLST.^2^, ^3^ Although this increase in threshold positivity has been reported to decrease false‐positive results,^7^, ^9^, ^10^ decision support tools and lung cancer risk prediction are still lacking for IPNs ≥6 mm. As such, we also performed size‐specific analysis based on three size classes of the nodules: <6 mm [small nodules], 6‐16 mm [intermediate‐sized nodules], and ≥16 mm [large nodules]. To our knowledge, this is one of the first radiomic analyses in lung cancer screening to utilize delta radiomic features (changes in radiomics over time) by nodule size class to predict lung cancer incidence.

## 2 MATERIALS AND METHODS

### 2.1 NLT study population

This research was approved by the Institutional Review Board (Advarra, Inc, Columbia, MD, USA). Deidentified data and LDCT images were obtained through the National Cancer Institute (NCI) Cancer Data Access System (CDAS).^9^ The NLST study design and main findings have been described previously.^2^, ^3^ Briefly, the NLST was a randomized multicenter trial comparing screening with LDCT to CXR in high‐risk individuals. Eligibility criteria included current or former smokers aged 55‐74 years with a minimum 30 pack‐years smoking history; former smokers had to have quit within the past 15 years.

### 2.2 NLST CT screening results

The NLST protocol defined a positive screening result as one or more noncalcified nodules or masses measuring ≥4 mm in axial diameter or, less commonly, other abnormalities such as adenopathy or pleural effusion.^2^, ^3^ Positive screens were defined in the setting of abnormalities on baseline screens or abnormalities on follow‐up screens that were new, stable, or that evolved with the latter demonstrating an increase in nodule size, consistency, or other characteristic potentially related to lung cancer. Participants with positive screening results received follow‐up recommendations; trial‐wide guidelines for the management of positive screens were developed, but were not mandated by protocol.

Negative screens were defined as CT scans with no abnormalities, minor abnormalities not suspicious for lung cancer, or significant abnormalities not suspicious for lung cancer. In this analysis, we did not include any participants who had a negative screening result.

### 2.3 Nested case–control study design

**Figure 1:**
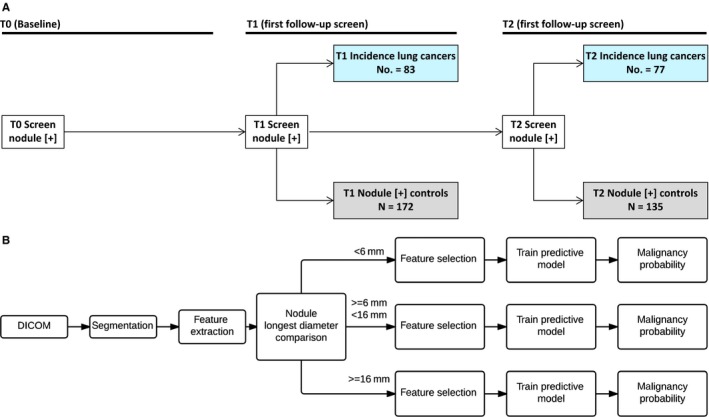
Schematic representations of the nested case–control study design (A) and the radiomics and analytical workflow (B)

We performed a nested case–control study comprised of screen‐detected incident lung cancers and matched nodule‐positive controls from the LDCT arm of the NLST. Based on the schema originally described in Schabath et al,^4^ the screen‐detected incident lung cancers and nodule‐positive controls are depicted in Figure 1A.

#### 2.3.1 Lung cancer cases

We identified 196 screen‐detected incident lung cancers who had a baseline‐positive screen (T0) that was not diagnosed as lung cancer and then were diagnosed at either the first (T1, N = 104) or second follow‐up (T2, N = 92).

#### 2.3.2 Nodule‐positive controls

Using a 2:1 to nested case–control study design, we identified 392 LDCT screening participants who had three consecutive positive screens (T0 to T2) that were not diagnosed as lung cancer. These NLST participants were designated as *nodule‐positive controls* in the current analysis. The nodule‐positive controls were frequency matched to the lung cancer cases’ age at enrollment (±5 years), sex, race/ethnicity, and smoking status. This study design minimizes the influence of confounders between the cases and the controls. As such, radiomic image features that differentiate cases and nodule‐positive controls are not likely be attributed to external risk factors.

#### 2.3.3 Training and test sets

Based on the availability of complete LDCTs and inability to verify the nodule/abnormality, the 192 lung cancer cases were reduced to 160. Likewise, the original set of 392 nodule‐positive controls was reduced to 307. The lung cases in cohort 1 were diagnosed at T1 and the lung cancer cases in cohort 2 were diagnosed at T2. All of the nodule‐positive controls had a positive scan from T0 to T2 and never developed lung cancer through T7 based on the available NLST data. Cohort 1 was used as a training set and Cohort 2 as a test set.

### 2.4 Target lung nodule identification

The identification of target lung nodules has been previously described.^11^ Briefly, two radiologists (YL and QL) reviewed all LDCT images at both the lung window setting (width, 1500 HU; level, −600 HU) and the mediastinal window setting (width, 350 HU; level, 40 HU). The identification of cancerous nodules among the screen‐detected incident lung cancers was based on data provided by the NLST (ie, location and size). As nodule location was not always available, the senior radiologist (YL)^11^ identified the nodules and manually mapped each nodule from T0 to T1. The locations of all nodules in this analysis are publically available in the TCIA database (www.cancerimagingarchive.net). For NLST participants with multiple lung nodules, the largest nodule at baseline (T0) and subsequent follow‐up nodule was used for radiomic feature extraction.

### 2.5 CT segmentation, feature extraction, and feature selection

The workflow of our radiomic pipeline^12^ and analyses is depicted in Figure 1B. As previously described,^11^ a single‐slick segmentation ensemble and subsequent feature extraction were performed using Definiens software (Definiens, Inc, AG Cambridge, MA, USA). There were 219 features extracted to quantify size, shape, location, and texture information of the pulmonary nodules.^6^ The complete list of features used in our analyses has been previously described^8^ and was reduced to the most consistent features based on our previous test/retest analyses. Additionally, we used features from the same filter that based on Cohort 1 were found to be “stable” over time (denoted as C1 stable). C1 stable features were filtered using an analogous approach to that for identifying RIDER stable features. For RIDER stable features, two LDCT screenings were performed in a 15‐minute interval. For the C1 stable features using the NLST subjects, we utilized T0 and T1 features as the test/retest set. For each feature, we computed the concordance correlation coefficient^13^ and dynamic range and we selected as C1 stable features those which had values for both parameters greater than 0.95. Even though we used a test/retest filter for initial feature selection, we built models which were able to classify data with the most predictive number of features. For that purpose, we used feature selectors ReliefF (RfF) and Correlation‐based Feature Selector (CFS). In each analysis, we selected the top 5 and top 10 ranked features. Tables 2 and 4 present the performance statistics based on the models with the best AUROC.

### 2.6 Baseline and delta features

For all available cases and controls, we extracted radiomic features from the T0 baseline screen and the T1 follow‐up screen. To assess changes in nodules after an approximately one‐year interval, we subtracted the T0 and T1 features to generate delta features. For all patients in our analysis, the median time from randomization to the T1 screen was 375 days (interquartile range = 360‐400 days). As such, the time interval to the T1 screen is relatively consistent for all subjects and eliminates the need to normalize the delta features with respect to time. In Tables 2 and 4, delta features are denoted with a “∆” and baseline features are denoted with “T0”.

### 2.7 Size‐specific analyses: Splitting the training and test sets on nodule size

Size‐specific analyses were performed based on the longest diameter (LD) of the T0 nodules. Current recommendations by the NCCN and the American College of Radiology (ACR) have been increased for a positive scan to have a 6 mm longest diameter nodule^5^ rather than the 4 mm originally used in the NLST.^3^ As such, we performed size‐specific analyses using three nodule size classes: <6 mm [small nodules], 6‐16 mm [intermediate‐sized nodule], and ≥16 mm [large nodules]. Because there were only 16 lung cancer cases and 7 nodule‐positive controls in the large size class (≥16 mm), we combined the intermediate and large class and repeated the analyses with two size classes: <6 mm [small] and ≥6 mm [large].

For computing overall accuracy, sensitivity, and specificity, we summarized confusion matrices of each size group and based on the result produce statistical parameters for the model. Computation of the area under the receiver operating characteristic (AUROC) uses a list of probabilities indicating an instance belongs to a class. For computation of the “overall” AUROC, we merged probability lists for each size group and produced the result on the final list.

### 2.8 Classifiers

Of the 219 features, there were 23 RIDER stable features and 37 C1 stable features. The C1 stable features are provided in Table S1. Features marked with asterisk symbol in Table S1 are used in RIDER stable feature set. Although we used a test/retest filter initial selection, our goal was to identify a model that is able to classify data with a small number of features. Size‐specific nodules from Cohort 1 were utilized to create the training dataset. For each training dataset, we applied a feature selector in order to simplify resulting model and remove noisy features. Selected features were used to train a classifier and after training on a corresponding subset of Cohort 2 used for testing. From multiple possible models, we selected the one which produces the highest AUROC. For the feature selectors, we used ReliefF (RfF)^14^, ^15^, ^16^ and Correlation‐based Feature Selector (CFS). For each feature selector, we selected the top 5 and 10 ranked features to identify highly predictive parsimonious models. One of the benefits we gained from splitting datasets is the independent usage of classifiers. For each subset, we applied the following classifiers:

- Decision tree—J48^17^;
- Rule‐based Classifier—JRIP^18^;
- Naive Bayes^19^;
- Support Vector Machine (SVM)^19^;
- Random Forests.^20^

For the SVM classifier, we utilized a radial basis function as a kernel and also a linear kernel. C and Gamma were found on the training set using Grid Search. Performance statistics and 95% confidence intervals (CIs) were calculated for each model including AUROC, accuracy, sensitivity, and specificity. All the experiments were performed in Weka version 3.6.13.^21^

### 2.9 Synthetic Minority Oversampling Technique

Because of the imbalance of case and controls across the various size classes, we also applied Synthetic Minority Oversampling Technique (SMOTE)^22^ in the analyses. SMOTE is an oversampling approach in which the minority class is over‐sampled by creating “synthetic” examples rather than by oversampling with replacement. To create a synthetic instance, one example (nodule feature vector) is randomly picked from minority class. For that example, five nearest neighbors in the same class are chosen. Then, one of these neighbors is randomly chosen. For each numeric feature, the example and its chosen neighbor produce a line segment between the two features. A new synthetic instance represents a randomly chosen point on the line segment for each feature. The process repeats with a new example randomly chosen until the desired number of instances is produced.

## 3 RESULTS

**Figure 2:**
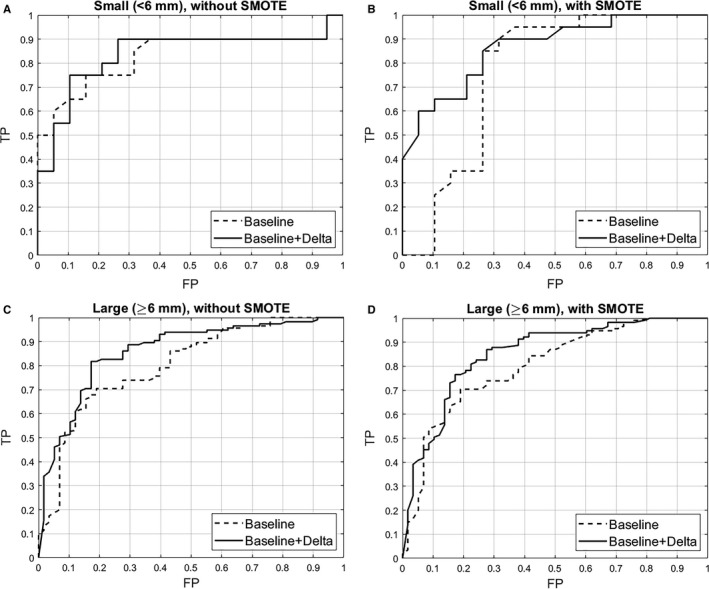
AUROC figures for the final models for small nodules without SMOTE (A), small nodules w ith SMOTE (B), large nodules without SMOTE (C), and large nodules with SMOTE (D)

The study population characteristics for the three size classes by the training and test sets of the lung cancer cases and nodule‐positive controls are presented in Table 1. None of the study population characteristics were significantly different between the training cohort and test cohort (Table S2) and, as previously reported (Table 1 in^11^), none of the study population characteristics are significantly different between the lung cancer cases and nodule‐positive controls. The final models for the three nodule size classes (Table 2 and Figure 2A‐D) generally revealed modest improvements in the performance statistics for models with baseline and delta radiomic features vs. models with only baseline radiomics. The AUROC for small‐sized nodules was 0.83 (95% CI 0.76‐0.90) for baseline‐only radiomic features and 0.84 (95% CI 0.77‐0.90) for baseline and delta features. For intermediate‐sized nodules, the AUROC was 0.76 (95% CI 0.71‐0.81) for baseline‐only radiomic features and 0.84 (95% CI 0.80‐0.88) for baseline and delta features. For large‐sized nodules, the AUROC was higher for baseline‐only radiomic features (AUROC = 0.86; 95% CI 0.75‐0.91) compared with baseline and delta features (AUROC = 0.83; 95% CI 0.75‐0.91).

**Table 1:** Study population characteristics of incident lung cancer cases and nodule‐positive controls by three nodule size classes

Training set (C1)	Lung cancer cases			Nodule‐positive controls		
	Small (<6 mm) N = 14	Intermediate (6‐16 mm) N = 53	Large (≥16 mm) N = 16	Small (<6 mm) N = 40	Intermediate (6‐16 mm) N = 125	Large (≥16 mm) N = 7
Age, mean (SD)	66.4 (4.8)	63.4 (5.1)	66.3 (5.6)	64.1 (5.2)	64.0 (5.3)	62.4 (5.1)
Sex, N (%)						
Male	9 (64.3)	27 (50.9)	8 (50.0)	23 (57.5)	76 (60.8)	4 (57.1)
Female	5 (35.7)	26 (49.1)	8 (50.0)	17 (42.5)	49 (39.2)	3 (42.9)
Race, N (%)						
White	14 (100.0)	49 (92.5)	16 (100.0)	39 (97.5)	120 (96.0)	6 (85.7)
Non‐White	0 (0.00)	4 (7.6)	0 (0.00)	1 (2.5)	5 (4.0)	1 (14.3)
Smoking status, N (%)						
Former	8 (57.1)	24 (45.3)	10 (62.5)	16 (40.0)	63 (50.4)	5 (71.4)
Current	6 (42.9)	29 (54.7)	6 (37.5)	24 (60.0)	62 (49.6)	2 (28.6)
Pack‐years, mean (SD)	70.39 (27.8)	63.5 (23.7)	54.8 (13.1)	64.8 (28.3)	65.1 (25.1)	60.5 (19.2)
Family history of lung cancer						
No	8 (57.1)	4 (79.3)	11 (68.8)	33 (82.5)	104 (83.2)	5 (71.4)
Yes	6 (42.9)	11 (20.8)	5 (31.3)	7 (17.5)	21 (16.8)	2 (28.6)
Stage						
I	6 (42.9)	42 (79.3)	12 (75.0)	—	—	—
II	3 (21.4)	5 (9.4)	0 (0.0)	—	—	—
III	1 (7.1)	2 (3.8)	4 (25.0)	—	—	—
IV	4 (28.6)	3 (5.7)	0 (0.0)	—	—	—
NOS	0 (0.00)	1 (1.9)	0 (0.00)	—	—	—
Histology						
Small cell	2 (14.3)	0 (0.0)	0 (0.0)	—	—	—
Adeno/BAC	8 (57.1)	35 (66.0)	14 (87.5)	—	—	—
Squamous cell	1 (7.1)	9 (17.0)	1 (6.3)	—	—	—
Other and NOS	3 (21.4)	9 (17.0)	1 (6.3)	—	—	—

Test set (C2)	Small (<6 mm) N = 19	Intermediate (6‐16 mm) N = 40	Large (≥16 mm) N = 18	Small (<6 mm) N = 20	Intermediate (6‐16 mm) N = 108	Large (≥16 mm) N = 7
Age, mean (SD)	63.4 (5.2)	62.6 (4.4)	63.3 (5.4)	61.2 (4.6)	63.1 (4.8)	63.9 (3.5)
Sex, N (%)						
Male	12 (63.2)	21 (52.5)	10 (55.6)	5 (25.0)	67 (62.0)	5 (71.4)
Female	7 (36.8)	19 (47.5)	8 (44.4)	15 (75.0)	41 (38.0)	2 (28.6)
Race, N (%)						
White	19 (100.0)	38 (95.0)	17 (94.4)	20 (100.0)	103 (95.4)	7 (100.0)
Non‐White	0 (0.0)	2 (5.0)	1 (5.6)	0 (0.0)	5 (4.6)	0 (0.0)
Smoking status, N (%)						
Former	9 (47.4)	19 (47.5)	9 (50.0)	9 (45.0)	47 (43.5)	4 (57.1)
Current	10 (52.6)	21 (52.5)	9 (50.0)	11 (55.0)	61 (56.5)	3 (42.9)
Pack‐years, mean (SD)	61.3 (32.4)	62.2 (21.5)	66.9 (24.2)	62.8 (21.9)	60.2 (20.9)	59.4 (21.4)
Family history of lung cancer						
No	13 (68.4)	34 (85.0)	12 (66.7)	18 (90.0)	91 (84.3)	5 (71.4)
Yes	6 (31.6)	6 (15.0)	6 (33.3)	2 (10.0)	17 (15.7)	2 (28.6)
Stage						
I	10 (52.6)	28 (70.0)	16 (88.9)	—	—	—
II	3 (15.8)	1 (2.5)	0 (0.0)	—	—	—
III	3 (15.8)	7 (17.5)	0 (0.0)	—	—	—
IV	3 (15.8)	3 (7.5)	2 (11.1)	—	—	—
NOS	0 (0.0)	1 (2.5)	0 (0.0)	—	—	—
Histology						
Small cell carcinoma	3 (15.8)	1 (2.5)	0 (0.0)	—	—	—
Adenocarcinoma/BAC	10 (52.6)	23 (57.5)	13 (72.2)	—	—	—
Squamous cell carcinoma	4 (21.1)	6 (15.0)	1 (5.6)	—	—	—
Other and NOS	2 (10.5)	10 (25.0)	4 (22.2)	—	—	—

**Table 2:** Final models for best AUROC by nodule size with three size classes for baseline‐only features and baseline plus delta features

Features	Baseline nodule size	Final models	AUROC (95% CI)	Accuracy (95% CI)	Specificity (95% CI)	Sensitivity (95% CI)
Baseline‐only	Small (<6 mm)	T_0_ Mean [HU] T_0_ Laws features L5 W5 L5 T_0_ Asymmetry T_0_ StdDev [HU] T_0_ Roundness	0.83 (0.76‐0.90)	0.59 (0.45‐0.72)	0.90 (0.78‐1.0)	0.26 (0.08‐0.44)
Baseline + Delta	Small (<6 mm)	T_0_ Mean [HU] T_0_ Is attached to pleural wall ∆ StdDev [HU] T_0_ Asymmetry T_0_ Circularity	0.84 (0.77‐0.90)	0.69 (0.57‐0.82)	0.90 (0.78‐1.0)	0.47 (0.27‐0.68)
Baseline‐only	Small (<6 mm) with SMOTE	T_0_ Mean [HU] T_0_ Asymmetry T_0_ MacSpic number of T_0_ Number of pixels T_0_ avgRLN T_0_ Volume [cm] T_0_ Volume (Pxl) T_0_ Short axis × Longest diameter T_0_ Longest diameter [mm] T_0_ Circularity	0.77 (0.69‐0.85)	0.77 (0.65‐0.88)	1.0 (1.0‐1.0)	0.53 (0.32‐0.73)
Baseline + Delta	Small (<6 mm) with SMOTE	T_0_ Mean [HU] T_0_ Attached to pleural wall ∆ StdDev [HU] T_0_ Asymmetry T_0_ Circularity T_0_ Roundness T_0_ Relative border to pleural wall T_0_ Relative border to lung T_0_ Volume (Pxl) T_0_ StdDev [HU]	0.86 (0.80‐0.92)	0.72 (0.59‐0.84)	0.95 (0.86‐1.04)	0.47 (0.27‐0.68)
Baseline‐only	Intermediate (6‐16 mm)	T_0_ Is attached to pleural wall T_0_ Mean [HU] T_0_ Longest diameter T_0_ Circularity T_0_ Roundness	0.76 (0.71‐0.81)	0.76 (0.70‐0.81)	0.92 (0.88‐0.97)	0.30 (0.18‐0.42)
Baseline + Delta	Intermediate (6‐16 mm)	T_0_ Is attached to pleural wall T_0_ Longest diameter ∆ max dist COG to border T_0_ Mean [HU] T_0_ Circularity	0.84 (0.80‐0.88)	0.80 (0.74‐0.85)	0.95 (0.92‐0.99)	0.37 (0.24‐0.50)
Baseline‐only	Intermediate (6‐16 mm) with SMOTE	T_0_ Is Attached To Pleural Wall T_0_ Mean [HU] T_0_ Longest diameter [mm] T_0_ Circularity T_0_ Roundness T_0_ Short axis × Longest diameter T_0_ Relative border to lung T_0_ Asymmetry T_0_ Relative border to pleural wall T_0_ StdDev [HU]	0.77 (0.72‐0.82)	0.82 (0.76‐0.87)	0.87 (0.82‐0.92)	0.68 (0.55‐0.80)
Baseline + Delta	Intermediate (6‐16 mm) with SMOTE	∆ Number of pixels ∆ SD Dist COG to border [mm] T_0_ MAX Dist COG to border [mm] ∆ Volume [cm ] T_0_ Volume (Pxl) ∆ Short axis × longest diameter T_0_ Circularity ∆ MAX Dist COG to border [mm] ∆ Mean [HU] ∆ avgRLN	0.85 (0.81‐0.89)	0.86 (0.81‐0.91)	0.97 (0.95‐1.0)	0.55 (0.42‐0.68)
Baseline‐only	Large (≥16 mm)	T_0_ SD dist COG to border T_0_ Volume [pix] T_0_ Circularity T_0_ Short axis × longest diameter T_0_ Max dist COG to border T_0_ Relative border to lung T_0_ Laws features E5 E5 R5 T_0_ Longest diameter T_0_ Roundness T_0_ Volume [cm]	0.86 (0.75‐0.91)	0.76 (0.77‐0.99)	0.62 (0.28‐0.97)	1.0 (1.0‐1.0)
Baseline + Delta	Large (≥16 mm)	T_0_ Mean [HU] T_0_ Laws features E5 E5 L5 T_0_ Relative border to lung T_0_ Relative border to pleural wall T_0_ Volume [pxl] ∆ Short axis [mm] T_0_ Laws features L5 W5 L5 ∆ Volume [pxl] T_0_ Is attached to pleural wall ∆ avgRLN	0.83 (0.75‐0.91)	0.88 (0.77‐0.99)	0.71 (0.36‐1.0)	0.94 (0.85‐1.0)
Baseline‐only	Large (≥16 mm) with SMOTE	T_0_ Mean [HU] T_0_ Longest diameter [mm] T_0_ Short axis × longest diameter T_0_ Short axis [mm] T_0_ StdDev [HU]	0.89 (0.82‐0.96)	0.80 (0.66‐0.94)	0.43 (0.04‐0.82)	0.94 (0.85‐1.04)
Baseline + Delta	Large (≥16 mm)—SMOTE	T_0_ Mean [HU] T_0_ Laws features E5 E5 L5 T_0_ Relative border to lung T_0_ Relative border to pleural wall T_0_ Volume (Pxl) ∆ Short axis [mm] T_0_ 3D laws features L5 W5 L5 ∆ Volume (Pxl) T_0_ Attached to pleural wall ∆ avgRLN	0.80 (0.71‐0.89)	0.80 (0.66‐0.94)	0.57 (0.18‐0.96)	0.89 (0.76‐1.02)
Baseline‐only	Overall[Link]		0.83 (0.82‐0.86)	0.74 (0.69‐0.79)	0.90 (0.86‐0.95)	0.45 (0.36‐0.55)
Baseline + Delta	Overall[Link]		0.86 (0.83‐0.89)	0.78 (0.74‐0.83)	0.93 (0.90‐0.97)	0.53 (0.44‐0.63)
Baseline‐only	Overall with SMOTE[Link]		0.81 (0.78‐0.84)	0.81 (0.76‐0.85)	0.87 (0.82‐0.92)	0.70 (0.61‐0.79)
Baseline + Delta	Overall with SMOTE[Link]		0.87 (0.84‐0.90)	0.83 (0.78‐0.87)	0.95 (0.92‐0.98)	0.61 (0.52‐0.70)

“Overall” includes all nodule sizes

We also computed the overall AUROC (Table 2), which included all nodule sizes, for baseline‐only features (AUROC = 0.83; 95% CI 0.82‐0.86) and baseline and delta features (AUROC = 0.86; 95% CI 0.83‐0.89). As such, we had a higher AUROC and accuracy for the large‐sized nodule model (0.86) compared with the overall model (0.83). When comparing the overall model to the intermediate‐sized nodule model, the overall model had higher AUROC, but the intermediate‐sized model had higher accuracy (0.76 vs 0.74) and specificity (0.92 vs 0.90). When comparing the overall model to the small‐sized nodule model, the AUROCs and specificities were identical for small‐sized nodules. The overall AUROC for three size classes for baseline and delta features was 0.86 (0.83‐0.89), which was higher than the AUROCs for the three size‐specific models. However, the large‐sized nodule model had a higher accuracy than the overall model (0.88 vs 0.78). Likewise, the intermediate‐sized nodule model had a higher accuracy than the overall model (0.80 vs. 0.78).

We also found when we applied the SMOTE method, which over‐samples the minority class creating synthetic minority class examples, some of the performance statistics improved (Table 2).

Because there were only 16 lung cancer cases and 7 nodule‐positive controls with large nodules (≥16 mm), we combined the intermediate‐ and large groups and repeated the analyses (Tables 3 and 4 and Figure 2A‐D). As such, when the intermediate‐sized nodules and large nodules were combined into a single group (≥6 mm), the AUROC for baseline‐only features was 0.80 (95% CI 0.76‐0.84) compared with an AUROC of 0.86 (95% CI 0.83‐0.89) for baseline and delta features. The AUROC for the overall model was identical for the large‐sized nodule model; however, the large‐sized nodule model has higher accuracy and specificity. Figure 2Aa‐C presents the AUROC plots for the final models for the small nodules and large nodules with and without SMOTE.

**Table 3:** Incident lung cancer cases and nodule‐positive controls by nodule size with by two nodule size classes

Training set (C1)	Lung cancer cases		Nodule‐positive controls	
	Small (<6 mm) N = 14	Large (≥6 mm) N = 69	Small (<6 mm) N = 40	Large (≥6 mm) N = 132
Age, mean (SD)	66.4 (4.8)	64.1 (5.3)	64.1 (5.2)	63.9 (5.3)
Sex, N (%)				
Male	9 (64.3)	35 (50.7)	23 (57.5)	80 (60.6)
Female	5 (35.7)	34 (49.3)	17 (42.5)	52 (39.4)
Race, N (%)				
White	14 (100.0)	65 (94.2)	39 (97.5)	126 (95.5)
Non‐White	0 (0.0)	4 (5.8)	1 (2.5)	6 (4.6)
Smoking status, N (%)				
Former	8 (57.1)	34 (49.3)	16 (40.0)	68 (51.5)
Current	6 (42.9)	35 (50.7)	24 (60.0)	64 (48.5)
Pack‐years, mean (SD)	70.3 (27.8)	61.5 (22.0)	64.8 (28.3)	64.8 (24.8)
Family history of lung cancer				
No	8 (57.1)	53 (76.8)	33 (82.5)	109 (82.6)
Yes	6 (42.9)	16 (23.2)	7 (17.5)	23 (17.4)
Stage				
I	6 (42.9)	54 (78.3)	—	—
II	3 (21.4)	5 (7.3)	—	—
III	1 (7.1)	6 (8.7)	—	—
IV	4 (28.6)	3 (4.4)	—	—
NOS	0 (0.00)	1 (1.5)	—	—
Histology				
Small cell	2 (14.3)	0 (0.0)	—	—
Adeno/BAC	8 (57.1)	49 (71.0)	—	—
Squamous cell	1 (7.1)	10 (14.5)	—	—
Other and NOS	3 (21.4)	10 (14.5)	—	—

Test Set (C2)	Small (<6 mm) N = 19	Large (≥6 mm) N = 58	Small (<6 mm) N = 20	Large (≥6 mm) N = 115
Age, mean (SD)	63.4 (5.2)	62.8 (4.7)	61.2 (4.6)	63.2 (4.7)
Sex, N (%)				
Male	12 (63.2)	31 (53.5)	5 (25.0)	72 (62.6)
Female	7 (36.8)	27 (46.5)	15 (75.0)	43 (37.4)
Race, N (%)				
White	19 (100.0)	55 (94.8)	20 (100.0)	110 (95.7)
Non‐White	0 (0.0)	3 (5.2)	0 (0.0)	5 (4.4)
Smoking status, N (%)				
Former	9 (47.4)	28 (48.3)	9 (45.0)	51 (44.4)
Current	10 (52.6)	30 (51.7)	11 (55.0)	64 (55.7)
Pack‐years, mean (SD)	61.3 (32.4)	63.6 (22.3)	62.8 (21.9)	60.1 (20.9)
Family history of lung cancer				
No	13 (68.4)	46 (79.3)	18 (90.0)	96 (83.5)
Yes	6 (31.6)	12 (20.7)	2 (10.0)	19 (16.5)
Stage				
I	10 (52.6)	44 (75.9)	—	—
II	3 (15.8)	1 (1.7)	—	—
III	3 (15.8)	7 (12.1)	—	—
IV	3 (15.8)	5 (8.6)	—	—
NOS	0 (0.0)	1 (1.7)	—	—
Histology				
Small cell carcinoma	3 (15.8)	1 (1.7)	—	—
Adenocarcinoma/BAC	10 (52.6)	36 (62.1)	—	—
Squamous cell carcinoma	4 (21.1)	7 (12.1)	—	—
Other and NOS	2 (10.5)	14 (24.1)	—	—

**Table 4:** Final models for best AUROC for large nodules (≥6 mm) for baseline‐only features and baseline plus delta features

Features	Baseline nodule size	Final model	AUROC (95% CI)	Accuracy (95% CI)	Specificity (95% CI)	Sensitivity (95% CI)
Baseline‐only	Large (≥6 mm)	T_0_ Mean [HU] T_0_ Roundness T_0_ Circularity T_0_ Is attached to pleural wall T_0_ Longest diameter T_0_ Max dist COG to border T_0_ StdDev [HU] T_0_ SD dist COG to border T_0_ 3D Laws features L5 W5 L5 T_0_ Relative border to lung	0.80 (0.76‐0.84)	0.75 (0.70‐0.80)	0.89 (0.85‐0.99)	0.46 (0.35‐0.58)
Baseline + Delta	Large (≥6 mm)	T_0_ Mean [HU] T_0_ Is attached to pleural wall ∆ Max dist COG to border ∆ Longest diameter T_0_ Roundness T_0_ Circularity ∆ Mean [HU] T_0_ SD dist COG to border T_0_ Longest diameter T_0_ Max dist COG to border	0.86 (0.83‐0.89)	0.82 (0.77‐0.87)	0.93 (0.89‐0.97)	0.60 (0.49‐0.71)
Baseline‐only	Large (≥6 mm) with SMOTE	T_0_ Mean [HU] T_0_ Roundness T_0_ Circularity T_0_ Is Attached to pleural wall T_0_ Longest diameter [mm] T_0_ MAX Dist COG to border [mm] T_0_ StdDev [HU] T_0_ SD Dist COG to border [mm] T_0_ 3D laws features L5 W5 L5 T_0_ Relative border to lung	0.79 (0.75‐0.83)	0.73 (0.67‐0.78)	0.83 (0.78‐0.89)	0.52 (0.41‐0.63)
Baseline + Delta	Large (≥6 mm) with SMOTE	T_0_ Mean [HU] T_0_ Attached to pleural wall ∆ MAX Dist COG to border [mm] ∆ Longest diameter [mm] T_0_ Roundness T_0_ Circularity ∆ Mean [HU] T_0_ SD Dist COG to border [mm] T_0_ Longest diameter [mm] T_0_ MAX Dist COG to border [mm]	0.85 (0.82‐0.88)	0.80 (0.75‐0.85)	0.87 (0.82‐0.92)	0.66 (0.55‐0.76)
Baseline‐only	Overall[Link]		0.80 (0.76‐0.83)	0.72 (0.67‐0.77)	0.90 (0.85‐0.94)	0.41 (0.32‐0.51)
Baseline + Delta	Overall[Link]		0.86 (0.83‐0.89)	0.8 (0.75‐0.84)	0.92 (0.89‐0.96)	0.57 (0.48‐0.67)
Baseline‐only	Overall with SMOTE[Link]		0.78 (0.75‐0.81)	0.74 (0.69‐0.79)	0.86 (0.81‐0.91)	0.52 (0.42‐0.61)
Baseline + Delta	Overall with SMOTE[Link]		0.85 (0.82‐0.88)	0.78 (0.74‐0.83)	0.88 (0.84‐0.93)	0.61 (0.52‐0.7)

“Overall” includes all nodule sizes

## 4 DISCUSSION

While lung cancer screening with LDCT for high‐risk individuals has unequivocally demonstrated that early detection saves lives, the current screening strategy comes at the identification of large numbers of indeterminate nodules and limited clinical decision tools to manage nodules.^23^ As such, we conducted a nested case–control analysis of the NLST to identify radiomic‐based models that predict lung cancer incidence. We utilized training and test sets of incident lung cancer cases and nodule‐positive controls to generate performance statistics of baseline‐only radiomic features vs. the combination of time‐varying delta radiomic features and baseline features. Additionally, analyses were conducted across three nodule size classes. Overall, we found that combining delta radiomics with baseline radiomics generally improved the performance statistics to predict lung cancer incidence when compared to using only baseline radiomic features. However, we note inconsistent results in some of the performance statistics when comparing the overall models, which were not size‐specific, to the size‐specific models. As such, our findings suggest there is a trade‐off in terms of performance using nodule size‐specific models vs. an overall model.

Previous studies have shown the utility of delta radiomic in lung cancer prognostication and therapy response,^24^, ^25^ and to the best of our knowledge, this is the first analysis to consider delta radiomics in the lung cancer screening setting. The modest improvements by including delta features with the baseline features suggest there were not substantial time‐varying differences from the baseline screen (T0) to the first follow‐up screen (T1) which occurred 12 months later. In our previous work^4^ that evaluated the screening histories and outcomes from T0 to T2 of the entire CT‐arm of the NLST, there were 6921 nodule‐positive controls at T0, then 4951 positive screens at T1 of which only 104 were diagnosed as lung cancer. As such, the majority of the nodules were either stable at T1 (N = 4951 nodule‐positive controls) or they resolved and were scored as a negative screen T1 (N = 1488 negative screens). So, the observed modest improvements in performance statistics of delta radiomics in the NLST warrant their further evaluation in other screening settings.

In our previous work using baseline‐only features in the NLST,^11^ a random forest classifier identified a model of 23 features that could predict nodules that would be diagnosed as lung cancer 1 year after baseline with an AUROC of 0.83 and 2 years after baseline with an AUROC of 0.75. Our current analysis differed from the previous work^11^ in many ways. First, the prior work identified a single model based on the best accuracy using only baseline features. In the current analysis, we included delta radiomics, generated radiomics models by nodule class size, trained our models to identify the features that achieved the best AUROCs, and we applied a SMOTE approach since there was an imbalance of case and controls across the various size classes. Additionally, to identify highly predictive parsimonious models with fewer features that were previously identified (23 features), we choose to identify models containing the top 5 and 10 features. We focused on AUROC because prior work demonstrated^26^ that AUROC is a better measure than accuracy in the evaluation of learning algorithms by demonstrating that AUROC is statistically consistent and more discriminating than accuracy.

**Figure 3:**
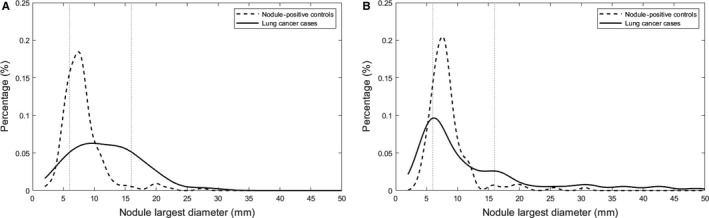
Histogram of longest diameter for the lung cancer cases and nodule‐positive controls for the training set (A) and test set (B)

A novel and important aspect on our analyses was the radiomic models by nodule size class. Nodule size is a key characteristic of malignancy whereby larger nodules have a higher probability of being diagnosed as lung cancer.^27^ As such, the management of nodules in current lung cancer screening guidelines is largely based on size and shape of the nodule.^5^, ^6^, ^7^ Certainly, reductions in false‐positive rates have been reported^7^, ^9^, ^10^ by increasing the size threshold for a positive scan from 4 to 6 mm. Results from the Dutch‐Belgian Lung Cancer Screening (NELSON) trial 28 reported that small nodules (<5 mm) have a 0.4% probability of lung cancer while intermediate‐sized nodules [5‐10 mm] have nearly 3 times the probability (1.3%) and require additional risk stratification. Large nodules [≥10 mm] have 15.2% lung cancer probability and receive an immediate diagnostic workup. Because of the distribution of nodule sizes among the cases and controls (Figure 3), we selected different nodule size cut‐points. Importantly, we note that each size class yielded different final models of radiomic features suggesting the potential importance of size‐specific biomarkers to improve nodule management.

Another novel approach and subsequent finding in our analysis were the improvements of sensitivity and specificity when we applied SMOTE.^29^ Classification analyses using class‐imbalanced data are biased in favor of the majority class, and the bias is even larger for high‐dimensional data where the number of variables greatly exceeds the number of samples.^29^ To address potential bias and imbalance, we applied SMOTE as this is a popular oversampling method that was originally proposed to improve random oversampling. In our analyses, we found that SMOTE tended to have marginal influence on the AUROCs; however, we observed consistent modest improvements in sensitivity and specificity when SMOTE was utilized when compared to the same size class where SMOTE was not utilized. This suggests SMOTE is not beneficial in improving discrimination classifiers, which has been previously reported by Blagus and Lusa,^29^ but improves the performance of the classifier in terms of sensitivity and specificity.

There are some limitations and some strengths of this analysis. Although Lung‐RADS^TM^ categories^10^ are commonly used in lung cancer screening, we opted to utilize categories based on longest diameter size. However, using this nested case–control approach, we did not have adequate representation across Lung‐RADS^TM^ categories^10^ since the majority of the nodules were between 6 and 16 mm. Nonetheless, our analyses did demonstrate that nodule size‐specific models may have utility in improving some performance statistics compared with an overall model. Another potential limitation is the nested case–control design resulting in the modest sample size. The nested design was utilized because it is not feasible to segment and extract radiomic features on >4,000 T0‐ and T1‐positive scans. Although our radiomic pipeline is well‐established^12^ and is efficient for studies on lung cancer screening, lung cancer outcomes, and radiogenomics,^11^, ^30^, ^31^, ^32^, ^33^, ^34^, ^35^, ^36^ nodule identification and segmentation is still a time‐consuming bottleneck. However, we are actively pursuing approaches for automated segmentation which will allow us to segment and extract radiomic features on large numbers of LDCT scans. We acknowledge there were fewer lung cancer cases in the training set and there was an imbalance across size classes; however, training on a subset improved accuracy and area under the AUROC to predict lung cancer incidence. Another possible limitation is that unmeasured/unknown cofounders may exist between the lung cancer cases and nodule‐positive controls. However, we attempted to reduce confounding between the lung cancer cases and nodule‐positive controls by matching on key demographic features. Despite the modest aforementioned limitations, we applied a rigorous training and testing analyses to identify informative, parsimonious models that predict lung cancer incidence in the lung cancer screening setting.

In conclusion, we demonstrated that the inclusion of delta radiomic features improves the ability to classify which lung nodules will be diagnosed as an incident lung cancer more accurately than previous reports.^37^, ^38^, ^39^, ^40^, ^41^ At present, adjunct biomarkers are not currently used for lung cancer screening, largely attributed to their early stage in development.^42^ Published reports have found that blood‐based and circulating biomarkers exhibited sensitivity values ranging from 40% to 91% and specificity values from 75% to 84%,^43^, ^44^, ^45^ with possible cancer detection capability as early as 12‐29 months prior to a lung cancer diagnosis.^46^ But, a critical goal of biomarker research is to add value to existing risk assessment standards, and the biomarker should be designed to supplement the current diagnostic/management tools.^47^ As such, radiomic‐based biomarkers are attractive because they can be incorporated into the current radiology workflow, are noninvasive, and can be generated from standard‐of‐care images negating the requirement of additional laboratory‐based biomarkers.

## CONFLICT OF INTEREST

Dr Schabath is an Associate Editor for *Cancer Medicine,* and the authors confirm that this does not alter their adherence to *Cancer Medicine* Editorial policies and criteria. Dr Gillies is a member of the Advisory Board for HealthMyne, Inc
