# Supplementary material for: Delta radiomic features improve prediction for lung cancer incidence: A nested case–control analysis of the National Lung Screening Trial
Source: Cancer Med. 2018 Dec 1;7(12):6340–56. doi: 10.1002/cam4.1852 (PMC6308046; doi:10.1002/cam4.1852)
Supplement: Supplementary file 1 [file CAM4-7-6340-s001.docx]

Supplemental Materials for:

**Delta radiomic features improve prediction for lung cancer incidence: A nested case-control analysis of the National Lung Screening Trial**

Dmitry Cherezov^1^, Samuel H. Hawkins^2^, Dmitry B. Goldgof^1^, Lawrence O. Hall^1^, Ying Liu^2,3^, Qian Li^2,3^, Yoganand Balagurunathan^2^, Robert J. Gillies^2^, Matthew B. Schabath^4*^

^1^Department of Computer Sciences and Engineering, University of South Florida, Tampa, Florida; ^2^Department of Cancer Physiology, H. Lee Moffitt Cancer Center and Research Institute, Tampa, Florida;

^3^Department of Radiology, Tianjin Medical University Cancer Institute and Hospital, National Clinical Research Center of Cancer, Key Laboratory of Cancer Prevention and Therapy; ^4^Department of Cancer Epidemiology, H. Lee Moffitt Cancer Center and Research Institute, Tampa, Florida

***Corresponding Author:** Matthew B. Schabath, Ph.D.

H. Lee Moffitt Cancer Center and Research Institute,

12902 Magnolia Drive MRC-CANCONT, Tampa, Florida 33612.

E-mail: [Matthew.Schabath@Moffitt.org](mailto:Matthew.Schabath@Moffitt.org)

| **Supplemental Table 1. Stable features in cohort 1** |
| --- |
| 1. Longest Diameter* 2. Short Axis x Longest Diameter* 3. Short Axis* 4. Mean* 5. Volume* 6. Is Attached To Pleural Wall* 7. Relative Border To Lung* 8. Relative Border To PleuralWall* 9. 3D Compactness 10. AV Dist COG To Border [mm] 11. SD Dist COG To Border [mm]* 12. MIN Dist COG To Border [mm] 13. MAX Dist COG To Border [mm]* 14. Relative Volume AirSpaces 15. Number AirSpaces 16. Av Volume AirSpaces [mm] 17. Compactness 18. Shape index 19. Area (Pxl) 20. Volume (Pxl)* 21. Number of pixels* 22. Width (Pxl) 23. Thickness (Pxl) 24. Length (Pxl) 25. Border length (Pxl) 26. avgGLN 27. avgHGRE 28. avgLRHGE 29. avgRLN* 30. avgRP 31. avgSRHGE 32. 3D Laws features L5 L5 L5 33. Histogram ENTROPY 34. Histogram SKEW 35. 3D Wavelet. P2 L2 C13 36. 3D Wavelet. P2 L2 C14 37. 3D Wavelet. P2 L2 C15 |
| **RIDER stable features* |

| **Supplemental Table 2. Comparison of study population characteristics between the training (C1) and test (C2) cohorts^1^** | | | |
| --- | --- | --- | --- |
| **Characteristic by cohort** | | **Lung Cancer Cases** | **Nodule Positive Controls** |
| **Age** | |  |  |
| C1 | mean (SD) | 64.4 (5.3) | 63.9 (5.2) |
| C2 | mean (SD) | 62.9 (4.8) | 62.9 (4.8) |
| P-value | | 0.06 | 0.06 |
|  |  |  |  |
| **Sex, N (%)** | |  |  |
| C1 | Female | 39 (46.7) | 69 (40.1) |
| C1 | Male | 44 (53.1) | 103 (59.9) |
| C2 | Female | 34 (44.2) | 58 (42.9) |
| C2 | Male | 43 (55.8) | 77 (57.1) |
| P-value | | 0.719 | 0.615 |
|  | |  |  |
| **Race, N (%)** | |  |  |
| C1 | White | 79 (95.2) | 165 (95.9) |
| C1 | Non-White | 4 (4.8) | 7 (4.1) |
| C2 | White | 74 (96.1) | 130 (96.3) |
| C2 | Non-White | 3 (3.9) | 5 (3.7) |
| P-value | | 0.775 | 0.870 |
|  | |  |  |
| **Smoking Status, N (%)** | |  |  |
| C1 | Former | 42 (50.6) | 84 (48.8) |
| C1 | Current | 41 (49.4) | 88 (51.2) |
| C2 | Former | 37 (48.1) | 60 (44.4) |
| C2 | Current | 40 (51.9) | 75 (55.6) |
| P-value | | 0.747 | 0.444 |
|  | |  |  |
| **Pack-years** | |  |  |
| C1 | mean (SD) | 63.0 (23.1) | 64.8 (25.6) |
| C1 | mean (SD) | 63.1 (24.9) | 60.5 (20.9) |
| P-value | | 0.986 | 0.113 |
|  | |  |  |
| **Family history of Lung Cancer** | |  |  |
| C1 | No | 61 (73.5) | 142 (82.6) |
| C1 | Yes | 22 (26.5) | 30 (14.4) |
| C2 | No | 59 (76.6) | 114 (84.4) |
| C2 | Yes | 18 (23.4) | 21 (15.6) |
| P-value | | 0.648 | 0.658 |
|  | |  |  |
| **Stage** | |  |  |
| C1 | Early Stage and NOS (I+II) | 68 (82.9) | -- |
| C1 | Late Stage (III+IV) | 14 (17.1) | -- |
| C2 | Early Stage and NOS (I+II) | 58 (76.3) | -- |
| C2 | Late Stage (III+IV) | 18 (23.7) | -- |
| P-value | | 0.302 |  |
|  | |  |  |
| **Histology** | |  | -- |
| C1 | Small cell | 2 (2.4) | -- |
| C1 | Adeno/BAC | 57 (68.7) | -- |
| C1 | Squamous cell | 11 (13.3) | -- |
| C1 | Other and NOS | 13 (15.7) | -- |
| C2 | Small cell | 4 (5.2) | -- |
| C2 | Adeno/BAC | 46 (59.7) | -- |
| C2 | Squamous cell | 11 (14.3) | -- |
| C2 | Other and NOS | 16 (20.8) | -- |
| P-value | | 0.587 |  |
| ^1^Student’s *t*-test was used to test for differences in continuous variables and Pearson chi-square test was used to test for differences in categorical variables. Column percentages total to 100% within each cohort for each characteristic. | | | |
